# HDAC4 regulates apoptosis in Acan‐Cre^ERT2^ ;HDAC4^d^ ^/d^ mice with osteoarthritis by downregulating ATF4

**DOI:** 10.1002/2211-5463.13965

**Published:** 2025-02-03

**Authors:** Jingrui Huang, Yukun Xu, Yujia Li, Yiming Pang, Xueting Ding, Raorao Zhou, Dan Liang, Xianda Che, Yuanyu Zhang, Chunfang Wang, Wenjin Li, Pengcui Li

**Affiliations:** ^1^ Shanxi Key Laboratory of Bone and Soft Tissue Injury Repair, Department of Orthopedics Second Hospital of Shanxi Medical University Taiyuan China; ^2^ Academy of Medical Sciences Shanxi Medical University Taiyuan China; ^3^ MOE Key Laboratory of Coal Environmental Pathogenicity and Prevention Shanxi Medical University Taiyuan China; ^4^ Department of Embryology, School of Basic Medical Sciences Shanxi Medical University Taiyuan China; ^5^ Animal Experiment Center Shanxi Medical University Taiyuan China; ^6^ Department of Stomatology Second Hospital of Shanxi Medical University Taiyuan China

**Keywords:** apoptosis, ATF4/CHOP signaling pathway, HDAC4, osteoarthritis, transgenic mouse

## Abstract

Several studies have previously shown that histone deacetylase 4 (HDAC4) can regulate endoplasmic reticulum stress‐induced apoptosis through the activating transcription factor 4 (ATF4)/CAAT/enhancer binding protein homologous (CHOP) signaling pathway, thereby affecting the progression of osteoarthritis (OA). The present study investigated the regulatory mechanism of HDAC4 in chondrocyte apoptosis in OA using Acan‐Cre^ERT2^;HDAC4^fl/fl^ gene knockout mice. Forty mice were divided into four groups: TM‐DMM group [tamoxifen (TM) injection at 2 months of age and destabilization of the medial meniscus (DMM) surgery at 3 months], TM‐sham group (TM injection at 2 months of age and sham surgery at 3 months), no TM‐DMM group (corn oil injection at 2 months of age and DMM surgery at 3 months) and no TM‐sham group (corn oil injection at 2 months of age and sham surgery at 3 months). Apoptosis and cartilage damage were assessed through imaging, histological analysis, immunohistochemistry, reverse transcriptase‐PCR and terminal deoxynucleotidyl transferase‐mediated dUTP nick end labeling staining. HDAC4 knockdown resulted in increased osteophyte formation, significant narrowing of the joint space and increased articular cartilage damage. Furthermore, expression levels of key apoptosis‐related markers, ATF4, CHOP, caspase‐3 and caspase‐9, were significantly higher in the TM groups than in their respective control groups. Taken together, our results suggest that HDAC4 deficiency leads to increased apoptosis induced by the ATF4/CHOP signaling pathway in the pathogenesis of OA. Therefore, upregulation of HDAC4 may represent a potential therapeutic strategy.

## Graphical abstract

Using the AcanCreERT2;HDAC4fl/fl genetic mouse model, we further investigated the role of histone deacetylase 4 (HDAC4) in chondrocyte apoptosis. We found that knocking down HDAC4 may be associated with chondrocyte apoptosis by upregulating the activating transcription factor 4 (ATF4)/CAAT/enhancer binding protein homologous (CHOP) signaling pathway. Upregulating HDAC4 could potentially serve as a therapeutic target for osteoarthritis.

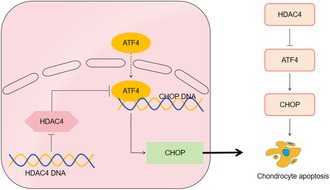

**Abbreviations**
ATF4: activating transcription factor 4
CHOP: CAAT/enhancer binding protein homologous protein
DMM: destabilization of the medial meniscus
ER: endoplasmic reticulum
HDAC: histone deacetylase
OA: osteoarthritis
OARSI: Osteoarthritis Research Society International
PBS: phosphate‐buffered saline
RT‐PCR: reverse transcriptase‐PCR
TM: tamoxifen
TUNEL: terminal deoxynucleotidyl transferase‐mediated dUTP nick end labeling

Osteoarthritis (OA) primarily affects individuals aged 65 years and older, and is the leading cause of knee dysfunction in this age group [1, 2]. Patients often experience pain and joint stiffness, which interfere with daily activities and significantly reduce their quality of life [3]. As a result of the limited blood supply and lack of effective neural control, the damage in affected joints is difficult to repair [4, 5]. Currently, the exact pathogenesis and molecular mechanisms of OA is still limited. There are no effective pharmacologic treatments available until the disease has progressed to the point where joint replacement is required.

Histone deacetylases (HDACs), also known as lysine deacetylases, regulate chromosome structure and gene expression through deacetylation modifications. They play a crucial role in biological processes such as cell proliferation, differentiation and apoptosis [6, 7]. Among them, HDAC4 has been increasingly implicated in processes like gene expression regulation, cell proliferation, differentiation, senescence and apoptosis. It is also involved in the onset and progression of various diseases, including osteosarcoma, gastric cancer and Huntington's disease [8, 9, 10, 11]. In the context of OA, HDAC4 is a key regulator of chondrocyte function and is considered one of the most studied HDACs in this field [12]. Research has shown that HDAC4 expression decreases during the progression of OA. *In vitro* experiments have demonstrated that overexpression of HDAC4 can reduce the levels of matrix metalloproteinase‐13, Indian hedgehog and type X collagen, at the same time as increasing the expression of type II collagen and aggrecan. These findings suggest that upregulation of HDAC4 may have a protective effect against the development of OA [13, 14].

Apoptosis, also known as programmed cell death, is a form of controlled, autonomous cell death [15]. This process plays a crucial role in maintaining the dynamic homeostasis of tissues, as well as in the growth and development of organisms. Dysregulation of apoptosis can lead to pathological conditions and contribute to the development of related diseases [16]. Recent studies have shown that apoptosis occurs in articular cartilage, with chondrocyte apoptosis being closely linked to the onset and progression of OA [17]. In normal cartilage, the proportion of cells undergoing apoptosis is typically only 2–5%. However, in osteoarthritic cartilage, this proportion can rise to as high as 18–21% [18]. Therefore, reducing chondrocyte apoptosis is important for regulating cartilage degeneration in OA [19].

Zhang *et al*. [20] found that HDAC4 inhibits the transcriptional activity of activating transcription factor 4 (ATF4) and the expression of its related target genes, such as CAAT/enhancer binding protein homologous protein (CHOP), through interaction with ATF4 using HEK293T cells. This inhibition reduces the expression of downstream pro‐apoptotic genes and decreases cell apoptosis. Similarly, Gu *et al*. [21] observed that HDAC4 expression was reduced in OA patients, whereas ATF4 and CHOP expression were elevated. *In vitro* experiments, where HDAC4 expression was upregulated or downregulated, showed corresponding decreases or increases in ATF4 and CHOP expression, along with a reduction or increase in chondrocyte apoptosis. Based on these findings, we hypothesize that HDAC4 may regulate chondrocyte apoptosis in cartilage by modulating ATF4. In the present study, we further investigated the role of HDAC4 in chondrocyte apoptosis and joint cartilage damage using Acan‐Cre^ERT2^;HDAC4^fl/fl^ gene knockout mice to reduce HDAC4 expression *in vivo*. Our results highlight HDAC4 as a critical regulatory factor in chondrocyte apoptosis, with significant theoretical and practical implications for understanding and treating OA.

## Materials and methods

### Transgenic mice

The animal protocol for this experiment was approved by the Laboratory Animal Ethics Committee of the Second Clinical Medical College of Shanxi Medical University (ethical filing number DW2022063). This approval complies with national regulations on the welfare and ethics of experimental animals and adheres to the Guidelines for the Care and Use of Laboratory Animals issued by the Chinese Society for Animal Research. Acan‐CreERT2 transgenic mice (stock no. 019148; Jackson Laboratory, Bar Harbor, ME, USA) [22] and HDAC4^fl/fl^ transgenic mice (purchased from Biocytogen, Beijing, China) were obtained [23, 24].

We mated Acan‐Cre^ERT2^ mice with HDAC4^fl/fl^ mice to produce Acan‐Cre^ERT2^;HDAC4^fl/fl^. A small portion of the mouse tails was collected, and DNA was extracted for genotyping to identify AcanCre^ERT2^;HDAC4^fl/fl^ mice. These mice were then maintained as previously described [25]. The biological characteristics of this transgenic mouse are similar to those of ordinary mice, and the mouse can grow and develop normally. However, after injection of tamoxifen (TM) dissolved in corn oil, the Acan‐CreERT2 in its body is activated, enabling the knockout of the HDAC4 gene with the fl/fl structure marker in cartilage tissue. This genotype allows for specific deletion of HDAC4 in cartilage tissue, particularly at the site of aggrecan expression [26, 27, 28]. Forty 2‐month‐old transgenic mice were randomly divided into two groups: one group received intraperitoneal injections of TM (100 μg·g^−1^·day^−1^) for 5 consecutive days to induce HDAC4 knockout (TM group, *n* = 20), whereas the other group received intraperitoneal injections of corn oil as a control (no TM group, *n* = 20). At 3 months of age, each group underwent either destabilization of the medial meniscus (DMM) surgery or sham surgery, resulting in four experimental groups (*n* = 10 per group): TM‐DMM, TM‐sham, no TM‐DMM and no TM‐sham. All mice were killed at 5 months of age.

The 40 mice were housed in a specific pathogen‐free barrier environment under a 12 : 12 h light/dark photocycle with controlled temperature and humidity. They were provided with a specific pathogen‐free diet and sterile drinking water. The health status of the mice was monitored daily, and records were taken every 3 days. Mice were killed using 100% CO_2_ when they reached any of the following humane endpoints: (a) weight loss (15–20% reduction from baseline weight); (b) anorexia (failure to eat for more than 24–48 h); (c) weakness (inability to eat or drink independently); and (d) infection (unresponsive to penicillin treatment). Death was confirmed by the absence of breathing for 2–3 min, lack of heartbeat and no blink reflex. During the course of the experiment, no unexplained deaths occurred.

Surgery was performed on the right knee joints of the mice and, at 5 months of age, all mice were killed. Following euthanasia, the right hind limbs were collected for further analysis. By comparing the outcomes of knockout and non‐knockout mice under both DMM and sham conditions, we aimed to gain further insight into the specific role and mechanisms of HDAC4 in the development of OA.

### DMM surgery

To induce traumatic OA in the DMM subgroup, we made a 0.5‐cm incision along the medial side of the patellar ligament with a sterile surgical scalpel, followed by hemostasis and layer‐by‐layer dissection. The patellar ligament was pushed laterally to expose the knee joint capsule, and then, under a microscope, the medial meniscotibial ligament was severed and meniscus was freed to confirm the success of the surgery. After DMM surgery, the medial displacement of the mouse knee joint reduced the weight‐bearing area, leading to increased pressure on the medial side. This mechanical alteration not only exacerbates stress on the inner knee joint, but also also accelerate the development of degenerative conditions, such as cartilage degeneration and osteophyte formation [29, 30]. The Sham group only underwent skin and joint capsule incision without any further intervention. Postoperatively, animals were allowed unrestricted movement with free access to food and water. The DMM surgery for all mice was performed by the same person on the same day.

### Imaging observations

The collected mouse hind limbs were X‐rayed with a small animal X‐ray machine (Faxitron Inc., Tucson, AZ, USA). Both anteroposterior and lateral views of the right hind limb were obtained. The anteroposterior scan was conducted to fully expose the knee joint space, while during the lateral scan, the knee joint curvature was maintained at approximately 150°. The exposure settings of the instrument were configured to the fully automatic default settings. According to previously published research, imaging can be classified into four levels [31, 32].

### Histological evaluation

The right hind limbs were fixed in 4% paraformaldehyde buffer for 48 h, then rinsed under running water for 30 min. The limbs were subsequently decalcified in 10% EDTA solution for 8 weeks, with the decalcification solution replaced every 5 days. After decalcification, the limbs were rinsed in running water for 2–3 h, then dehydrated using a fully automated dehydrator (Leica, Wetzlar, Germany) with a graded ethanol series. Finally, the specimens were embedded in paraffin, and 5‐μm thick sections were prepared in the coronal plane using a rotary slicer (Leica).

The sections were baked on a slide warmer (Leica) at 60 °C for 2 h, then sequentially immersed in xylene, absolute ethanol and 95% ethanol. Staining was performed using Safranin O‐Fast Green (Sigma‐Aldrich; Merck KgaA, Darmstadt, Germany). Two independent, blinded observers scored each section, and the scores were averaged for each joint according to the OARSI grading system [33]. The scoring system ranged from 0 to 6 points, with a higher score indicating more severe cartilage degeneration.

### Immunohistochemistry

Immunohistochemical staining was performed on paraffin sections to detect the expression of HDAC4, ATF4, CHOP, caspase‐3 and caspase‐9. After dewaxing and rehydration, endogenous peroxidase activity was blocked by incubating the sections with a blocking solution for 10 min, followed by three washes with phosphate‐buffered saline (PBS). Antigen retrieval was carried out using a complex enzyme (Boster, Wuhan, China), with incubation in a 37 °C oven for 1 h, followed by three PBS washes. The sections were then blocked with normal goat serum at room temperature for 30 min, and incubated overnight at 4 °C with the following primary antibodies: anti‐HDAC4 (dilution 1 : 50; catalog. no. A0179; Abclonal, Woburn, MA, USA), anti‐ATF4 (dilution 1 : 50; catalog. no. 10835‐1‐AP; Proteintech, Rosemont, IL, USA), anti‐CHOP (dilution 1 : 50; catalog. no. 15204‐1‐AP; Proteintech), anti‐caspase‐3 (dilution 1 : 50; catalog. no. bs‐0081R; BIOSS, Woburn, MA, USA) and anti‐caspase‐9 (dilution 1 : 50; catalog. no. bs‐0050R; BIOSS). After three PBS washes, a reaction enhancer was added dropwise and incubated for 30 min at 37 °C. Following another PBS wash, horseradish peroxidase‐conjugated secondary antibody (cat. no. PV‐9001; ZSGB‐BIO, Beijing, China) was applied and incubated in an oven at 37 °C for 1 h. Sections were then colorimetrically developed using 3,3′‐diaminobenzidine solution. Stained sections were viewed and photographed using a DM6B microscope (Leica) for subsequent quantitative analysis.

### Quantitative reverse transcriptase‐PCR (RT‐PCR)

Cartilage tissue was obtained from the knee joint of the right hind limb of mice in a mortar and grinded using liquid nitrogen. The right hind limbs of five mice were used in each group, and each mouse was an independent sample. To the ground tissue, 1 mL of TRIzol reagent (Invitrogen, Waltham, MA, USA) was added for RNA extraction, and the concentration of extracted RNA was measured using a NanoDrop Onec (Thermo Fisher Scientific, Waltham, MA, USA). RNA was reverse transcribed into cDNA using PrimeScript™ RT Master Mix (Takara). cDNA after reverse transcription was amplified using SYBR Green™ Premix Ex Taq™ II Amplification Kit (Takara, Shiga, Japan) using the following amplification procedure: pre‐denaturation (95 °C for 30 s), denaturation (95 °C for 15 s), annealing (60 °C for 45 s) and annealing (60 °C for 45 s). The amplification program was pre‐denaturation (95 °C for 30s), denaturation (95 °C for 15 s), annealing (60 °C for 45 s), for a total of 40 cycles; at the end of the amplification, the gene expression was calculated by the relative quantification of gene $2^{-\Delta \Delta C_{t}}$ method. The primers used in PCR can are shown in Table 1.

**Table 1:** Primers used in RT‐PCR.

Species	Genes	Forward	Reversed
Mice	HDAC4	5′‐CTGCAAGTGGCCCCTACAG‐3′	5′‐CTGCTCATGTTGACGCTGGA‐3′
	ATF4	5′‐TCGATGCTCTCTGTTTCGAATG‐3′	5′‐ATTTTCAGCTGGTCCAACGG‐3′
	CHOP	5′‐CTGCCTTTCACCTTGGAGAC‐3′	5′‐CGTTTCCTGGGGGATGAGATA‐3′
	Caspase‐3	5′‐TGGGACTGATGAGGAGA‐3′	5′‐ACTGGATGAACCACGAC‐3′
	Caspase‐9	5′‐GCCAGAGGTTCTCAGACCAG‐3′	5′‐TCCCTGGAACACAGACATCA‐3′
	18S	5′‐CGGCTACCACATCCAAGGAA‐3′	5′‐CGGCTACCACATCCAAGGAA‐3′

### Terminal deoxynucleotidyl transferase‐mediated dUTP Nick end labeling (TUNEL)

The paraffin sections were permeabilized with 0.1% Triton‐X‐100 in PBS at room temperature for 15–20 min. TUNEL staining was performed according to the instructions provided in the TUNEL Cell Apoptosis Detection Kit‐CY3 (catalog. no. MK1016; Boster). After the staining procedure, an anti‐fade mounting medium was applied to the sections. Fluorescence images were then captured using a fluorescence microscope.

### Statistical analysis

All experimental data are presented as the mean ± SD. Statistical analysis was performed using spss software (IBM Corp., Armonk, NY, USA) and prism, version 9.0 (GraphPad Software Inc., San Diego, CA, USA). Comparisons between two groups were made using a *t*‐test, whereas one‐way analysis of variance was used to compare data across four groups. Each experiment was repeated at least three times. *P* < 0.05 was considered statistically significant.

## Results

### Successful preparation of Acan‐Cre^ERT2^ ;HDAC4^fl^ ^/fl^ transgenic mice

**Fig. 1:**
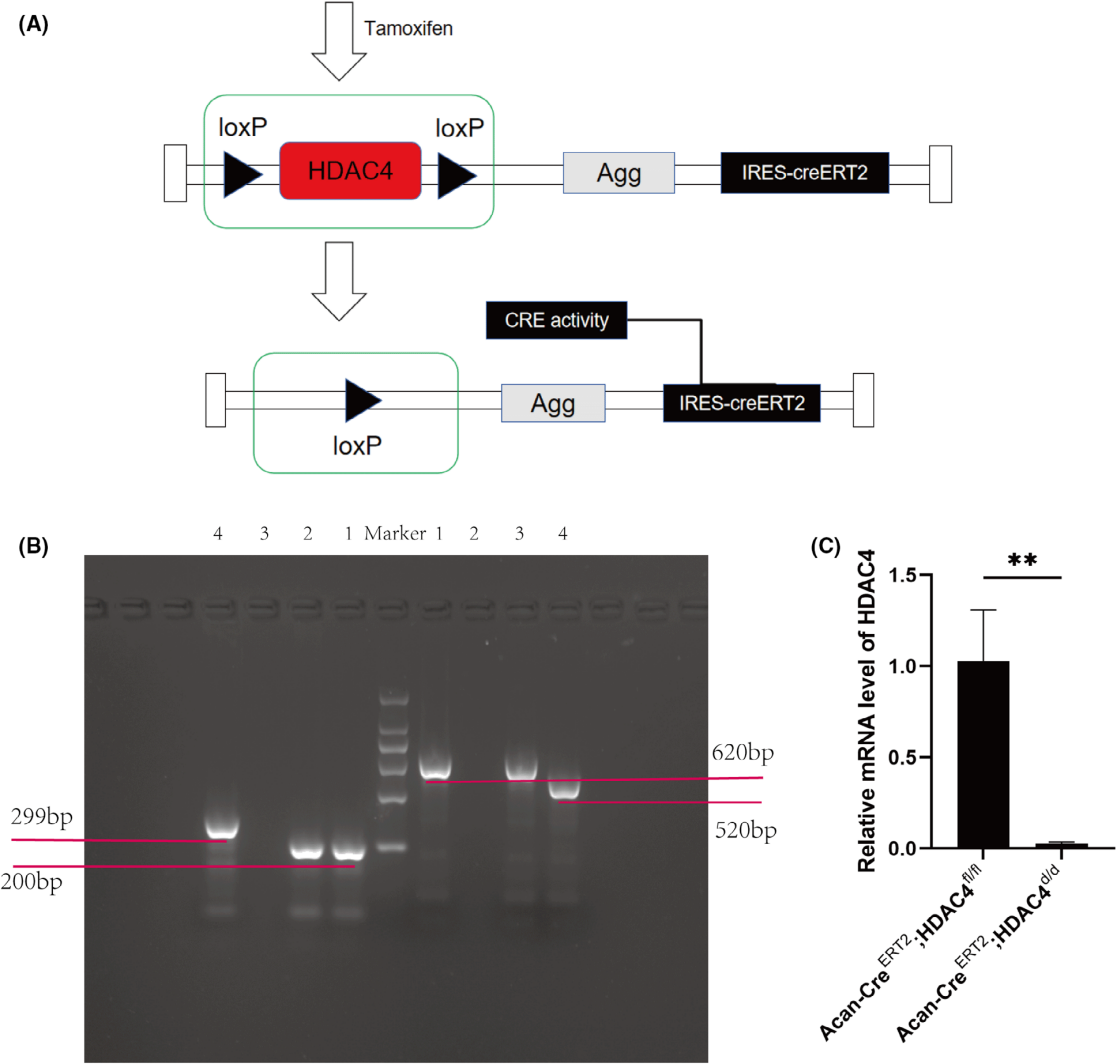
Construction of Acan‐Cre^ERT2^;HDAC4^fl/fl^ conditional HDAC4 knockout mice. (A) Illustration of Acan‐Cre^ERT2^;HDAC4^fl/fl^ mice. (B) DNA gel electrophoresis results of mouse tail identification: Channel 1: homozygous aggrecan and HDAC4 genes and is the mouse used in our experiment. Channel 2: homozygous aggrecan gene. Channel 3: homozygous HDAC4 gene. Channel 4: wild‐type mouse. (C) Tissue samples from tamoxifen‐injected genetically engineered mice (Acan‐Cre^ERT2^;HDAC4^d/d^) and non‐tamoxifen‐injected genetically engineered mice (Acan‐Cre^ERT2^;HDAC4^fl/fl^) were collected and analyzed for HDAC4 knockdown or not by quantitative RT‐PCR. All experimental data are presented as the mean ± SD. Each experiment was repeated three times. A *t*‐test was used to compare the two groups. **P < 0.01.

In this experiment, the Acan‐Cre^ERT2^; HDAC4^fl/fl^ transgenic mice were injected with TM, which activated the Cre recombinase enzyme. This activation resulted in the recombination of the loxP sites, leading to the knockout of the HDAC4 gene (Fig. 1A). Tail biopsy analysis confirmed the presence of the HDAC4 allele and the Acan‐Cre transgene (Fig. 1B). Channel 1 shows the PCR results from the transgenic mice used in this study. The homozygous aggrecan gene appears at 200 bp, and the homozygous HDAC4 gene appears at 620 bp. These data indicate that the transgenic mice are homozygous for both the aggrecan and HDAC4 alleles. After tamoxifen injection, RT‐PCR analysis revealed a significant reduction in HDAC4 gene expression in the knockout mice (Fig. 1C). These results demonstrate the successful generation of HDAC4 knockout mice.

### HDAC4 knockdown resulted in osteocphytes and significant narrowing of the joint space

**Fig. 2:**
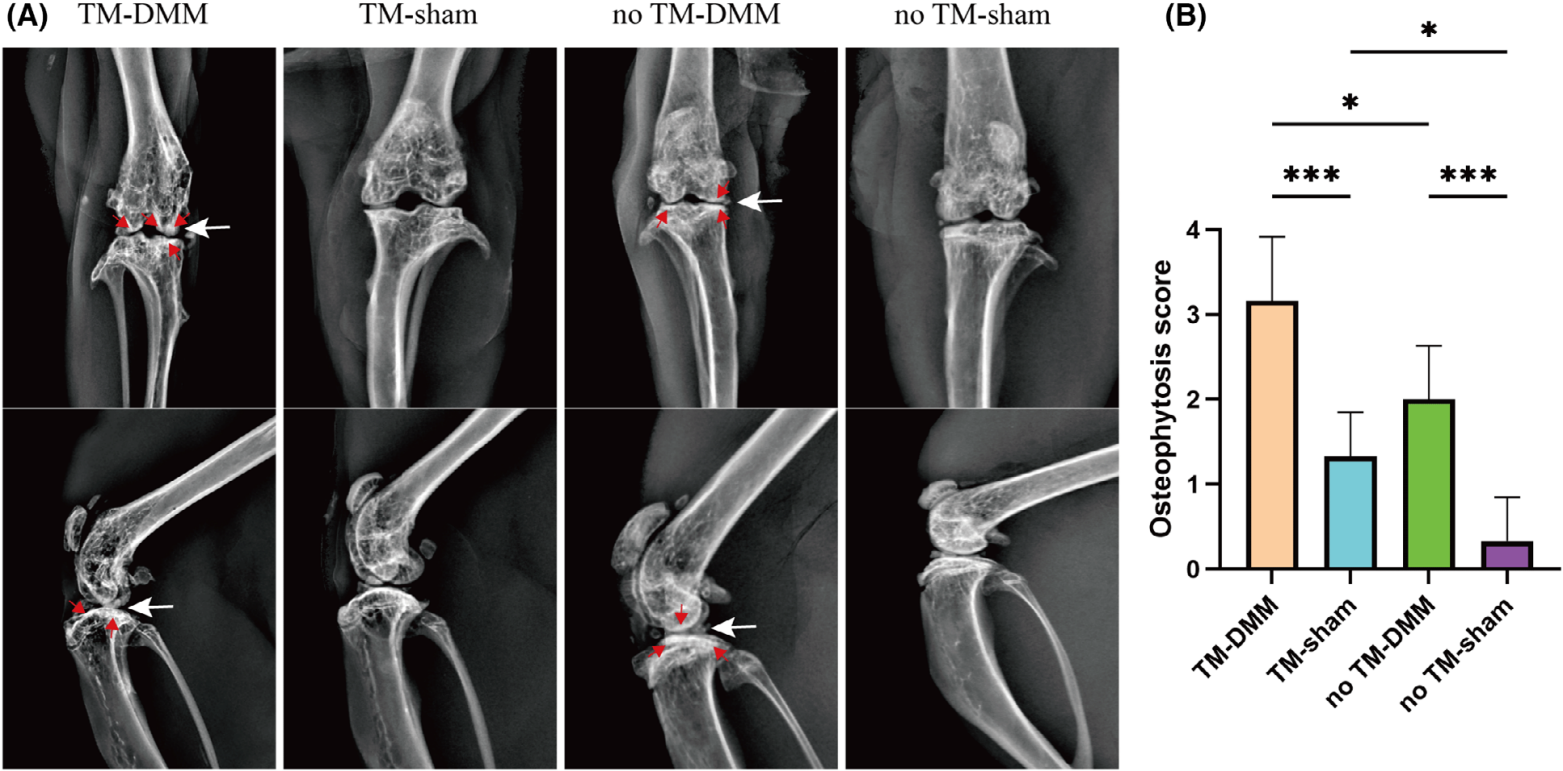
X‐ray analysis of the knee joints of mice 2 months after DMM surgery. (A) Compared with mice injected with corn oil, HDAC4 knockout DMM mice had a narrowed joint space (white arrows) and significant osteophyte (red arrows) formation. DMM, destabilization of the medial meniscus; TM, tamoxifen. (B) Osteophytosis scores in the X‐rays. All experimental data are presented as the mean ± SD. Each experiment was repeated three times. One‐way analysis of variance was used to compare the four groups. *P < 0.05 and ***P < 0.001.

X‐rays of the collected right hind limbs were performed 8 weeks after DMM surgery. X‐rays of the TM‐DMM group showed significant narrowing of the joint space, increased subchondral bone density and significant osteophytes in this group. No TM‐DMM group had an uneven joint surface, and there was minimal osteophyte formation with narrowing of the joint space, which was mild compared to TM‐DMM group. No obvious abnormality was seen in sham group (Fig. 2A). TM‐DMM osteophyte scores were higher than the other three groups (Fig. 2B).

### Knockdown of HDAC4 resulted in increased damage to articular cartilage

**Fig. 3:**
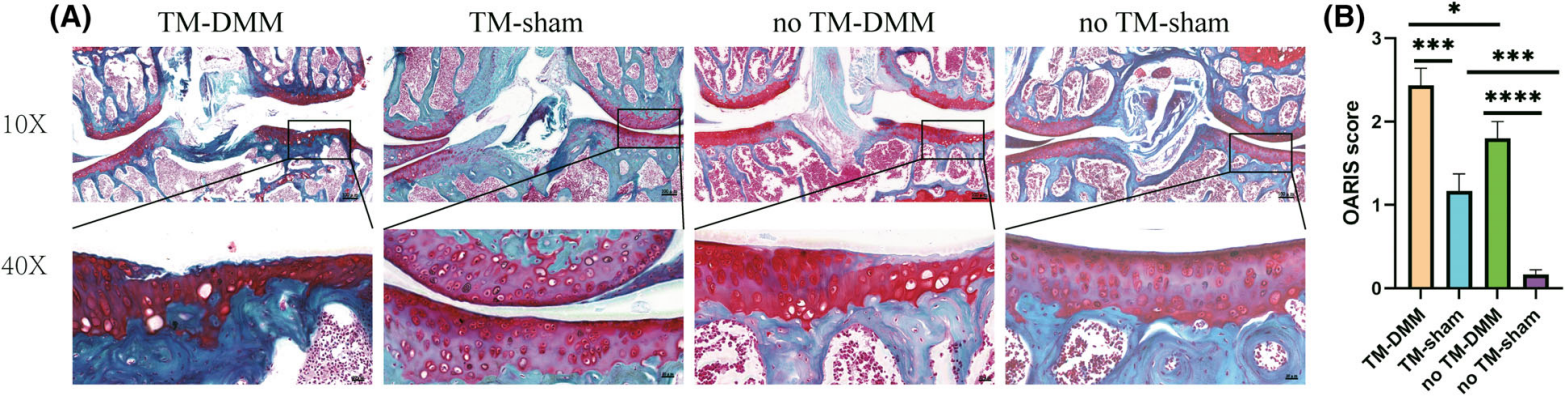
Knockdown of HDAC4 resulted in more cartilage damage. (A) Safranin O staining in the Acan‐Cre^ERT2^;HDAC4^d/d^ + DMM group showing extensive staining loss and severe cartilage damage. Top panel: scale bars = 100 μm. Bottom panel: scale bars = 20 μm. (B) OARSI scores. DMM, destabilization of the medial meniscus; HDAC4, histone deacetylase 4; OARIS, osteoarthritis research society international; TM, tamoxifen. All experimental data are presented as the mean ± SD. Each experiment was repeated three times. One‐way analysis of variance was used to compare the four groups. *P < 0.05, ***P < 0.001 and ****P<0.0001.

Based on Safranin O‐Fast Green staining (Fig. 3A), Acan‐CreERT2; HDAC4fl/fl conditional HDAC4 knockout mice exhibited more severe cartilage damage in their knee joints compared to the control group (no TM) 2 months after undergoing DMM surgery. The extent of cartilage damage was further assessed using the Osteoarthritis Research Society International (OARIS) scoring system, with the TM‐DMM group showing significantly higher OARIS scores than the no TM‐DMM group (Fig. 3B). Additionally, we observed an increased OARIS score in the TM‐sham group compared to the no TM‐sham group, suggesting that HDAC4 knockdown contributes to cartilage damage even in the absence of surgical intervention.

### Knockdown of HDAC4 resulted in elevated expression of ATF and CHOP, as well as increased apoptosis

**Fig. 4:**
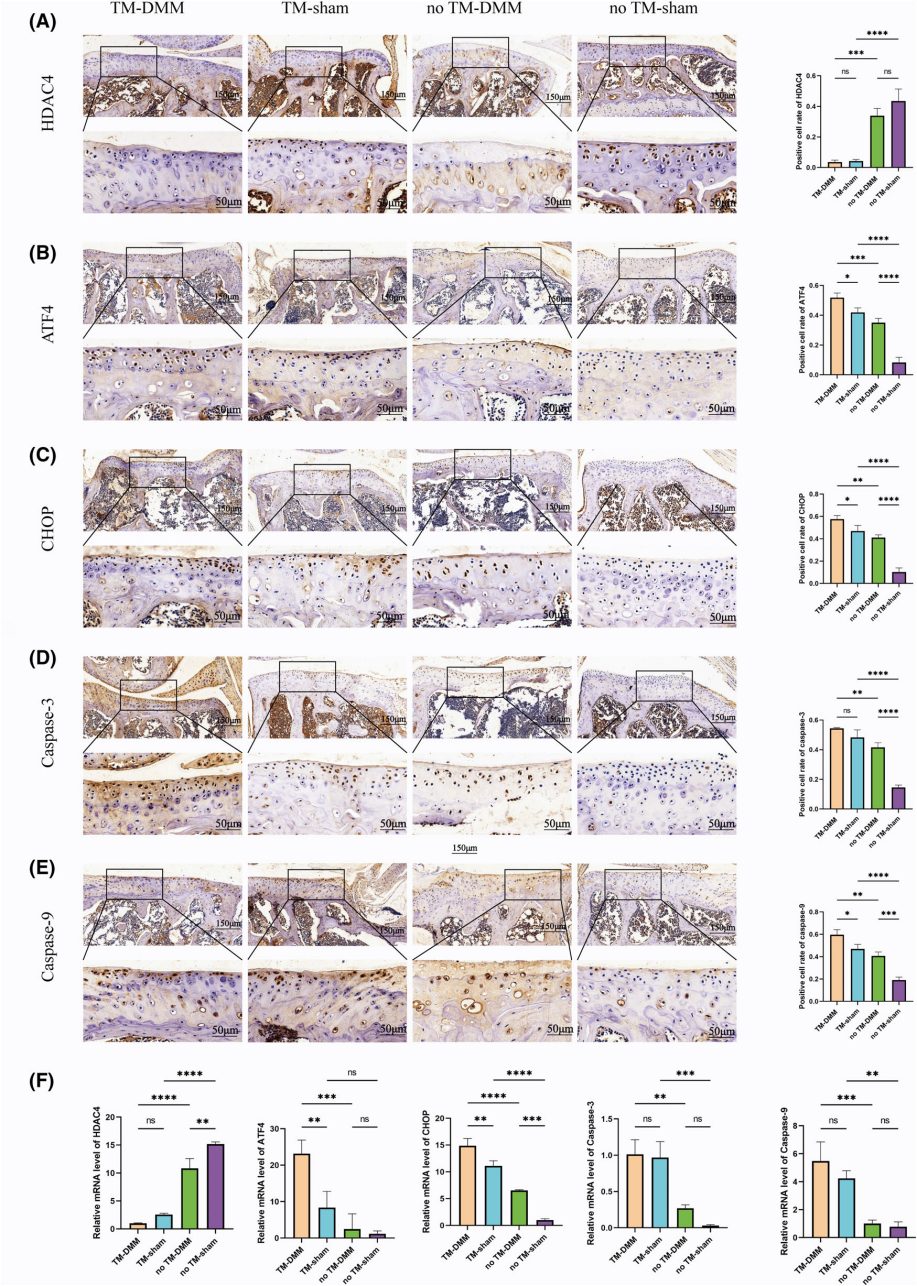
Immunohistochemical staining and RT‐PCR for the expression of HDAC4, ATF4, CHOP, caspase‐3 and caspase‐9 in transgenic murine osteoarthritis model. (A–E) Immunohistochemical staining showed HDAC4, ATF4, CHOP, caspase‐3 and caspase‐9 in 8‐week‐old DMM and sham transgenic mice and quantitative analysis of immunohistochemical staining. Top: scale bar = 150 μm. Bottom: scale bar = 50 μm. (F) RT‐PCR for HDAC4, ATF4, CHOP, caspase‐3 and caspase‐9 expression in 2‐month‐old DMM and sham transgenic mice. ATF4, activating transcription factor 4; CHOP, C/EBP homologous protein; DMM, destabilization of the medial meniscus; HDAC4, histone deacetylase 4; RT‐PCR, reverse transcription‐quantitative polymerase chain reaction; TM, tamoxifen. All experimental data are presented as mean ± standard deviation (mean ± SD). Each experiment was repeated three times. One‐way analysis of variance was used to compare the four groups. *P < 0.05, **P < 0.01, ***P < 0.001 and ****P<0.0001, ns, not significant.

**Fig. 5:**
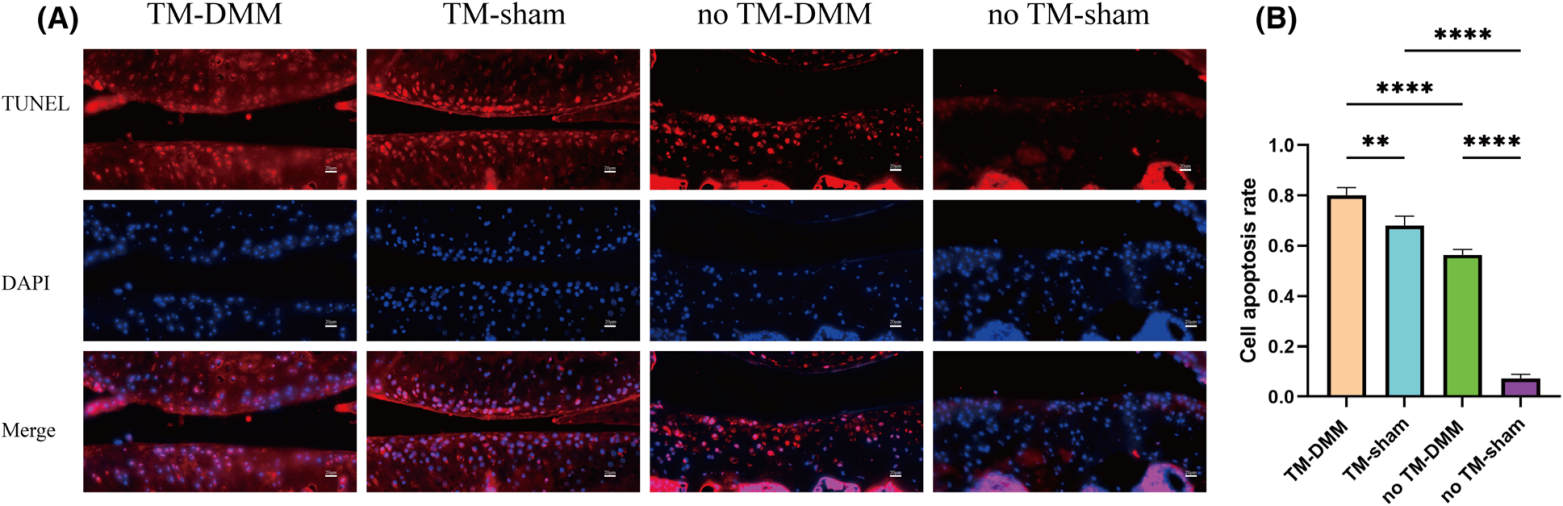
Decrease of HDAC4 resulted in increased apoptosis in Acan‐Cre^ERT2^;HDAC4^fl/fl^ gene mice. (A) TUNEL staining results showed in chondrocyte apoptosis. Scale bar = 20 μm. (B) Quantitative analysis of TUNEL. Staining showed that highest positive rate of apoptosis in the TM‐DMM group, followed by moderate in TM‐sham and no TM‐DMM, and the lowest rate in the no TM‐sham group. DMM, destabilization of the medial meniscus; TM, tamoxifen; TUNEL, terminal deoxynucleotidyl transferase‐mediated dUTP nick end labeling. All experimental data are presented as the mean ± SD. Each experiment was repeated three times. One‐way analysis of variance was used to compare the four groups. **P < 0.01, ***P < 0.001 and ****P<0.0001.

To investigate the impact of HDAC4 knockdown on apoptosis, we assessed the expression of key apoptosis‐related proteins using immunohistochemistry. In the sham group, TM injection led to a reduction in HDAC4 expression and a concomitant increase in the expression of ATF4, CHOP, caspase‐3 and caspase‐9. Notably, the TM‐DMM group exhibited a significantly higher expression of these markers compared to both the no TM‐DMM and sham groups, further supporting the role of HDAC4 in regulating apoptosis in articular cartilage (Fig. 4A–E). Additionally, TUNEL staining confirmed these findings, showing a higher chondrocyte positivity rate in the TM‐DMM group compared to both the no TM‐DMM and sham groups, indicative of increased apoptosis in the cartilage following HDAC4 knockdown (Fig. 5).

In addition, RT‐PCR analysis further validated the immunohistochemical results. In both the DMM and sham groups, the expression of HDAC4 was lower in the TM groups compared to the no TM groups. Conversely, the expression levels of ATF4, CHOP, caspase‐3 and caspase‐9 were significantly higher in the TM groups than in their respective no TM groups (Fig. 4F).

## Discussion

In the present study, we successfully established the Acan‐Cre^ERT2^;HDAC4^fl/fl^ mouse model using knockout technology to investigate the role of HDAC4 in the regulation of apoptosis in OA following DMM. OA and apoptosis were assessed through radiological imaging, Safranin O‐Fast Green staining, immunohistochemistry and TUNEL assays. X‐ray analysis of knee joints from HDAC4 knockout mice showed joint space narrowing and the formation of osteophytes on the tibial plateau and femoral condyles compared to mice without HDAC4 knockout. Safranin O‐Fast Green staining revealed severe cartilage damage in the HDAC4 knockout mice. Additionally, both immunohistochemistry and RT‐PCR analyses demonstrated significantly increased expression of apoptosis markers caspase‐3 and caspase‐9 in the HDAC4 knockout group. TUNEL staining further confirmed a significant increase in apoptosis in this group compared to the other three groups. These findings clearly highlight the critical role of HDAC4 in regulating apoptosis during OA in this mouse model.

The results of the present study also show that, in the TM‐sham group, although X‐ray imaging did not reveal joint space narrowing or osteophyte formation compared to the normal group, there was an increase in the OARIS score and elevated expression of ATF4/CHOP. This suggests that OA may be developing, even in the absence of overt radiographic changes. These findings are consistent with previous studies indicating that reduced HDAC4 accelerates OA progression, whereas upregulation of HDAC4 may exert a protective effect against OA. In the no TM‐DMM group, compared to the normal group, although HDAC4 immunohistochemistry results showed no statistically significant difference, a downward trend in HDAC4 expression was observed. RT‐PCR analysis further demonstrated a decrease in HDAC4 expression. Additionally, levels of ATF4/CHOP and apoptosis‐related markers were elevated, suggesting a link between cartilage damage and chondrocyte apoptosis. These findings imply that the elevated apoptosis markers may contribute to a detrimental feedback loop, exacerbating cartilage degeneration and further promoting OA progression [19, 34].

HDAC4 is expressed with notable tissue specificity, primarily in the brain, muscle, and cartilage [35, 36]. It has been shown to play a crucial role in the development of OA by inhibiting chondrocyte hypertrophy and maintaining cartilage integrity [37, 38]. Apoptosis is a key factor in degenerative diseases, including OA, because chondrocyte apoptosis disrupts the balance between cartilage synthesis and degradation, leading to increased damage to the cartilage matrix [17, 39]. Previous studies have demonstrated that HDAC4 is predominantly localized in the cytoplasm, where it interacts with ATF4 to regulate its activity. Through this interaction, HDAC4 inhibits the expression of ATF4 and its downstream target genes, thereby suppressing apoptotic processes [20]. In the present study, we utilized Acan‐Cre^ERT2^;HDAC4^fl/fl^ conditional knockout mice to reduce HDAC4 expression, resulting in increased levels of ATF4 and CHOP. This elevation in ATF4 and CHOP was associated with a corresponding increase in chondrocyte apoptosis, further highlighting the importance of HDAC4 in cartilage homeostasis.

HDAC4 induces chondrocyte apoptosis by regulating the ATF4/CHOP pathway. ATF4 is a critical transcription factor involved in several key biological processes, including protein synthesis, amino acid metabolism and cellular responses to oxidative stress [40]. Furthermore, ATF4 activates the expression of another transcription factor, CHOP. Previous studies have demonstrated that mice lacking CHOP exhibit significant protection against endoplasmic reticulum (ER) stress‐induced apoptosis, indicating that CHOP plays a vital role in promoting apoptosis by inhibiting anti‐apoptotic signaling and regulating the cell cycle and metabolism [41, 42, 43, 44, 45]. The roles of ATF4 and CHOP in initiating ER stress processes, particularly how they activate ER stress signaling pathways to induce apoptosis, have been well established [46]. Our findings are consistent with these previous reports. We observed that knockdown of HDAC4 in an osteoarthritis model led to increased levels of ATF4 and CHOP, resulting in enhanced chondrocyte apoptosis.

Gu *et al*. [21] previously demonstrated that the expression of HDAC4 is reduced in OA cartilage, whereas the levels of ATF4, CHOP, caspase12 and apoptotic cells are elevated. In their study, modulation of HDAC4 expression, either through overexpression or suppression, led to corresponding changes in the levels of ATF4, CHOP, caspase12 and apoptosis. In an anterior cruciate ligament transection rat model, the injection of an HDAC4 adenovirus showed that HDAC4 can inhibit apoptosis induced by the ATF4/CHOP signaling pathway, thus mitigating joint cartilage damage. These findings suggest that HDAC4 exerts an anti‐apoptotic effect by blocking the ATF4/CHOP pathway, offering a potential therapeutic target for osteoarthritis treatment. Consistent with the previous findings of our research group [21], we can observe in this study that, when HDAC4 is knocked out, the expression levels of ATF4, CHOP, caspase‐3, caspase‐9 and apoptotic cells are all increased. In the present study, we used Acan‐Cre^ERT2^;HDAC4^fl/fl^ knockout mice for the first time to investigate the role of HDAC4 in regulating chondrocyte apoptosis and ATF4 in OA. The HDAC4 knockout mice specifically target the deletion of HDAC4 in chondrocytes, allowing for a more precise examination of the long‐term effects of HDAC4 loss on chondrocyte apoptosis and OA progression [26, 47]. This approach avoids the confounding factors related to injection‐induced injury that could potentially influence the results. By contrast, adenoviral injections generally produce short‐term effects and may be subject to variability as a result of infection efficiency and immune responses, which could introduce discrepancies in the results [48, 49]. Compared to adenoviral injection in rat knee joints, the HDAC4 knockout mouse model provides a more robust demonstration of the reliability of our findings. Additionally, the use of Acan‐Cre^ERT2^;HDAC4^fl/fl^ knockout mice addresses the gap in the *in vivo* studies by Gu, where the downregulation of HDAC4 was not fully examined, thus enhancing the persuasiveness of our conclusions, thereby strengthening the validity and significance of our conclusions.

In conclusion, our data indicate that decreased HDAC4 may be associated with chondrocyte apoptosis via upregulation of ATF4/CHOP pathway. Thus, upregulation of HDAC4 may be a new potential therapy for OA treatment.

## Conflicts of interest

The authors declare that they have no conflicts of interest.

### Peer review

The peer review history for this article is available at https://www.webofscience.com/api/gateway/wos/peer‐review/10.1002/2211‐5463.13965.

## Author contributions

JH, YX and PL designed the project. JH prepared the manuscript. JH, XD and RZ performed the experiments. JH and XC create the animal model. CW and PL revised the manuscript. All authors contributed to data analysis and integration, visualization and figure generation, and approved the final version of the manuscript submitted for publication.

## Data Availability

The data that support the findings of this study are available from the corresponding author upon reasonable request.
